# Lessons from SARS and MERS remind us of the possible therapeutic effects of implementing a siRNA strategy to target COVID‐19: Shoot the messenger!

**DOI:** 10.1111/jcmm.15652

**Published:** 2020-07-17

**Authors:** Solomon Habtemariam, Ioana Berindan‐Neagoe, Cosmin Andrei Cismaru, Dedmer Schaafsma, Seyed Fazel Nabavi, Saeid Ghavami, Maciej Banach, Seyed Mohammad Nabavi

**Affiliations:** ^1^ Pharmacognosy Research Laboratories and Herbal Analysis Services University of Greenwich Kent UK; ^2^ Research Center for functional Genomics, Biomedicine and Translational Medicine “Iuliu Hatieganu” University of Medicine and Pharmacy Cluj‐Napoca Romania; ^3^ Department of Functional Sciences, Immunology and Allergology “Iuliu Hatieganu” University of Medicine and Pharmacy Cluj‐Napoca Romania; ^4^ Science Impact Winnipeg MB Canada; ^5^ Applied Biotechnology Research Center Baqiyatallah University of Medical Sciences Tehran Iran; ^6^ Division of Translational Medicine, Baqiyatallah Hospital Baqiyatallah University of Medical Sciences Tehran Iran; ^7^ Department of Human Anatomy and Cell Sciences Rady Faculty of Health Sciences Max Rady College of Medicine University of Manitoba Winnipeg Canada; ^8^ Department of Hypertension Medical University of Lodz Lodz Poland; ^9^ Polish Mother's Memorial Hospital Research Institute (PMMHRI) Lodz Poland

Dear Editor,

Since its first identification as a human pathogen in the Wuhan province of China in December 2019, the SARS‐CoV‐2 virus, which causes COVID‐19, has become a global pandemic with immense medical and socio‐economic costs. Like other coronaviruses, such as severe acute respiratory syndrome (SARS) and Middle East respiratory syndrome coronavirus (MERS‐CoV), SARS‐CoV‐2 is a single‐stranded positive‐sense RNA virus. The SARS‐COV‐2 as well as the SARS‐COV and MERS‐COV genomes contain several open‐reading frames (ORFs) that play an essential role in viral pathogenicity and infection.^1^, ^2^, ^3^ Based on previous experiences with other coronaviruses, ORFs are considered to be essential for viral replication through encoding viral replicase proteins to synthesize mRNAs of subgenomic length.^1^, ^3^ Silencing or small/short interfering RNA (siRNA) is a gene silencing approach using a small fragment of approximately 20‐25 base pairs of double‐stranded RNA that binds to a specific site of the relevant/target messenger RNA (mRNA); siRNAs are designed to silence genes at the post‐transcriptional level (by inducing cleavage and subsequent degradation of target mRNA) and can therefore be considered as vaccines or therapeutic agents. Zheng et al^4^ designed 48 siRNA sequences that potentially target the entire SARS‐CoV genome RNA, including ORFs for the translation of several key proteins. Among these, four siRNAs that could inhibit SARS‐CoV infection in foetal rhesus monkey kidney cells (FRhK‐4), both in a prophylactic and post‐infection therapeutic manner, were identified. Translating this idea to live animal experiments, Li et al^5^ demonstrated a similar efficacy of siRNAs in a rhesus macaque (*Macaca mulatta*) SARS model. These agents, with no visible signs of toxicity, were shown to improve several symptoms of SARS‐CoV, such as fever, viral load and acute alveolar damage. Importantly, the efficacy of the siRNAs was evident at relatively small respiratory doses (10‐40 mg/kg). Similar experiments have been performed with different siRNA sequences targeting various regions of the SARS‐CoV genome. For example, He et al^6^ showed that miRNAs targeting the replicase 1A region were more effective against the virus in FRhK‐4 cells. In 293 and HeLa cells, siRNAs targeting SARS‐CoV RNA‐dependent RNA polymerase (RDRP) showed therapeutic potential as well by specifically inhibiting RDRP expression.^7^ In addition, this system reduced plaque formation in Vero‐E6 cells, a cell line classically used to identify and count hemorrhagic fever viruses. In these cells, siRNA to target and inhibit gene expression of SARS‐CoV spike (S) protein has been successfully utilized *in vitro*.^8^, ^9^ Similarly, siRNAs efficiently targeting S protein coding regions have been identified using FRhk‐4 cells and an *in vivo* rhesus macaque model of SARS‐CoV infection.^10^ In line with these findings, the aforementioned efficacy of the siRNA developed by Li et al^5^ was based on the S protein coding and ORF1b (NSP12) regions. Envelope (E) and membrane (M) proteins could also be (specifically) targeted, as demonstrated in SARS‐CoV‐infected FRhk‐4 cells.^11^ In addition to synergistic effects that may be exhibited by different siRNAs, their therapeutic action can synergize with other currently existing antiviral agents through direct or indirect targeting common structural genes or other cellular targets.^11^, ^12^

In principle, several proteins encoded by the viral genome can be targeted by siRNA technology.^13^ He et al^14^ demonstrated the power of synergistic antiviral effects through siRNA targeting of various structural genes such as S, envelope, membrane and nucleocapsid. An additional advantage of siRNA technology is the incredibly low dose required to eliminate SARS‐CoV infection; for example, less than 60 nmol/L in Vero E6 cells^15^ and 10‐40 mg/kg/daily in monkeys was sufficient for satisfactory therapeutic effects.^5^ The application and potential effectiveness of siRNAs have also been evaluated in MERS‐CoV using computational models.^16^ In view of angiotensin‐converting enzyme 2 (ACE2) as a recognized host cell receptor for the SARS‐CoV S protein, the development of siRNAs targeting key host proteins could hold promise. Indeed, silencing ACE2 expression in Vero E6 cells by siRNA (containing sequences homologous to a section of ACE2) significantly reduced SARS‐CoV infection.^17^

Overall, the described studies on the effectiveness of specific siRNAs to battle SARS‐CoV and MERS‐CoV provide sufficient rationale to at least consider the use of siRNA strategies to target the closely related virus SARS‐CoV‐2. Despite their promising therapeutic effects, the application of higher doses of siRNAs, if so required, may be associated with some challenges, including adaptive^18^ and innate immune responses,^19^, ^20^ unwanted target effects, and saturation of the endogenous small RNA machinery.^21^ It is comforting, however, that previous data from several randomised, double‐blind, placebo‐controlled trials indicate that ALN‐RSV01 (a siRNA‐based drug) is safe to use and effective against respiratory syncytial virus infection.^22^, ^23^ Taken together, siRNA‐based therapeutics might be considered as an effective strategy to treat of COVID‐19. Future studies are warranted to evaluate their potential efficacy and safety.

## CONFLICT OF INTEREST

The authors declare no competing interests.

## AUTHOR CONTRIBUTION

**Solomon Habtemariam:** Formal analysis (equal); Investigation (equal). **Ioana Berindan‐Neagoe:** Conceptualization (equal); Investigation (equal). **Cosmin Andrei Cismaru:** Formal analysis (equal); Methodology (equal). **Dedmer Schaafsma:** Investigation (equal); Methodology (equal). **Seyed Fazel Nabavi:** Conceptualization (equal); Data curation (equal). **Saeid Ghavami:** Investigation (equal); Methodology (equal). **Maciej Banach:** Investigation (equal); Methodology (equal). **Seyed Mohammad Nabavi:** Conceptualization (equal); Formal analysis (equal).

1

**FIGURE 1:**
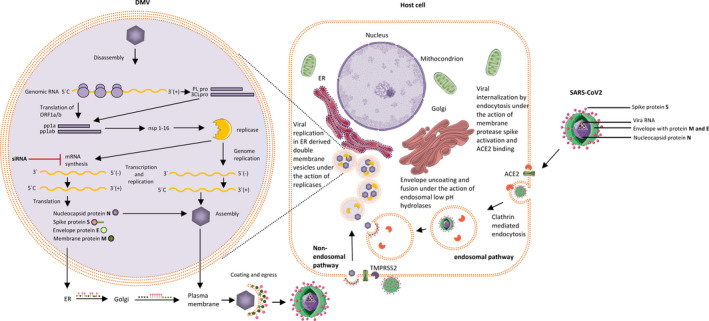
Potential effects of siRNAs on silencing viral genes at the post‐transcriptional level in COVID‐19. Coronaviruses enter the cell via the endosomal pathway exploiting autophagy or the non‐endosomal pathway, both leading to the release of the nucleocapsid into the cytoplasm. Replication of genomic RNA takes place in double‐membrane vesicles (DMVs) shielded from host immune responses, where the translation of ORF1a/b into the replicase polyprotein 1a (pp1a) and pp1ab will take place. Papain‐like proteases (PLpro) and 3C‐like protease (3CLpro) cleave pp1a and pp1ab to produce non‐structural proteins (nsp), including replicases (RNA‐dependent RNA polymerases) and helicases. The positive‐strand genomic RNA is transcribed to form a negative‐strand template for the synthesis of new genomic RNAs and subgenomic negative‐strand templates. mRNA is synthesized and translated into producing the structural and accessory viral proteins. siRNAs can potentially silence genes at post‐transcriptional level, degrading mRNA and blocking its translation. (adapted after Zumla et al^24^)
